# Beta-Glucan From Barley Attenuates Post-prandial Glycemic Response by Inhibiting the Activities of Glucose Transporters but Not Intestinal Brush Border Enzymes and Amylolysis of Starch

**DOI:** 10.3389/fnut.2021.628571

**Published:** 2021-04-16

**Authors:** Lovemore Nkhata Malunga, Nancy Ames, Haonan Zhouyao, Heather Blewett, Sijo Joseph Thandapilly

**Affiliations:** ^1^Richardson Center for Functional Foods and Nutraceuticals, Agriculture and Agri-Food Canada, Winnipeg, MB, Canada; ^2^Department of Food and Human Nutritional Sciences, University of Manitoba, Winnipeg, MB, Canada; ^3^Morden Research and Development Centre, Agriculture and Agri-Food Canada, Morden, MB, Canada; ^4^Canadian Centre for Agri-Food Research in Health and Medicine (CCARM), St Boniface Hospital Research Centre, Winnipeg, MB, Canada

**Keywords:** barley, beta-glucan, post-prandial glucose response, viscosity, GLUT2, SGLT1, *in vitro* digestion

## Abstract

Beta (β)-glucan (BG) from cereal grains is associated with lowering post-prandial blood glucose but the precise mechanism is not well-elucidated. The main aim of this study was to understand the mechanism through which BG from barley affects post-prandial glycemic response. Waffles containing 0, 1, 2, and 3 g barley BG and the same amount of available carbohydrate (15 g) were fed to the TIM-1 dynamic gastrointestinal digestion system to study the effect of BG on starch hydrolysis. Intestinal acetone powder and *Xenopus laevis* oocytes were used to study BG's effect on mammalian intestinal α-glucosidase and glucose transporters. The presence of BG did not significantly affect the *in vitro* starch digestion profiles of waffles suggesting that BG does not affect α-amylase activity. Intestinal α-glucosidase and glucose transport activities were significantly (*p* < 0.0001) inhibited in the presence of barley BG. Interestingly, BG viscosity did not influence α-amylase, α-glucosidase, GLUT2, and SGLT1 activities. This study provides the first evidence for the mechanism by which BG from barley attenuates post-prandial glycemic response is *via* alteration of α-glucosidase, GLUT2, and SGLT1 activity, but not amylolysis of starch. The decrease in post-prandial blood glucose in the presence of BG is likely a consequence of the interaction between BG and membrane active proteins (brush border enzymes and glucose transporters) as opposed to the commonly held hypothesis that increased viscosity caused by BG inhibits starch digestion.

## Introduction

Several human feeding studies have shown that beta (β)-glucan (BG) from barley can mitigate the rise in post-prandial glycemic response after consuming a high available carbohydrate meal, but the results are not consistent (1–3). These inconsistencies have been attributed to the changes in concentration, type of BG, or the BG to available carbohydrate ratio present in the food matrix. The precise mechanism through which BG attenuates glycemic response remains a subject of investigation. The widely hypothesized mechanism through which cereal BG reduces post-prandial blood glucose is its ability to form viscous solutions (4, 5). It is purported that BG increases luminal viscosity thereby decreasing the interaction between digestive enzymes and their substrates as well as simple sugars and intestinal nutrient transport proteins (6, 7). This has been supported by the evidence that high molecular weight BG tends to be more effective in impairing intestinal carbohydrate assimilation than its low molecular weight counterparts (8). BG viscosity, like the viscosity of most soluble fibers, is a function of its molecular weight and concentration when the solubility, pH and temperature are the same (9, 10). Thus, grain processing or meal preparation techniques that compromise BG molecular weight are thought to be responsible for the lack of effect in some BG human feeding trials (11). The drawback of the hypothesis related to viscosity is that the viscosity of soluble fiber is highly susceptible to shear thinning (12) and thus BG viscosity may be drastically reduced or nullified in the presence of the intestinal peristaltic force during digestion (13).

In human studies, intake of ≤ 3.5 g of BG per day does not significantly affect post-prandial blood glucose concentration but ≥ 6 g BG per day for at least 4 weeks does (14). However, for a single meal effect, the ratio of BG to available carbohydrates in the meal appears to be critical to BG's efficacy in lowering post-prandial blood glucose. For example, in one study, a BG to starch ratio of 0.16 was more effective in impairing starch digestibility than that of 0.11 (10). Most human studies suggest that 4 g or more of BG for every 30 g of available carbohydrates is required to notice a significant decrease in post-prandial blood glucose. Consequently, EFSA approved a health claim for barley and oat BG and post-prandial blood glucose when the dose is ≥ 4 g BG for every 30 g available carbohydrate (15). In a recent study conducted by our group (Clinicaltrial.gov Identifier: NCT02367989) which aimed to understand the effect of BG dosage and ratio to available carbohydrate, all three doses tested (2, 4, and 6 g of BG per 30 g of available carbohydrate) were effective in reducing post-prandial glycemic response with more than 20%, which is of physiological relevance. However, we did not observe any significant changes between 2, 4, or 6 g of BG per 30 g of available carbohydrate (16).

In theory, assimilation of available carbohydrate begins with the breakdown of starch by the saliva and pancreatic α-amylase in the mouth and the small intestine, respectively (17). The released maltose or α-limit dextrin together with disaccharides present in the food are hydrolysed to monosaccharides by the brush border maltase-glucoamylase and sucrase-isomaltase (18). The monosaccharides are later transported either passively through the intestinal nutrient transporter GLUT2 and GLUT5 or actively through the SGLT1 (19, 20). BG is likely to affect the activity of gastrointestinal carbohydrase or nutrient transporters for a reduced post-prandial blood glucose concentration (21). Thus, this study was specifically designed to understand the mechanism through which BG affects available carbohydrate digestibility, glucose bio-accessibility and uptake. Specifically, we evaluated the effect of BG on a) α-amylase activity using a computer-controlled dynamic multi-compartmental *in vitro* digestive platform (TIM-1 system), b) α-glucosidase activity using mammalian intestinal α-glucosidase, and c) glucose uptake activity using oocytes expressing either human GLUT2 or SGLT1. Additionally, we aimed to evaluate the test food we used in our recent human feeding study and to precisely understand the digestive mechanism of the BG containing test foods using the dynamic *in vitro* digestion platform.

## Materials and Methods

### Materials

Enzymes and bile for use in TIM-1 and α-glucosidase experiments were obtained from Sigma-Aldrich (St. Louis, MI, USA). Barley BG was obtained from Megazyme International Ireland [Bray, Wicklow, Ireland). Radiolabelled glucose ([3H] 2-deoxyglucose (25.5 Ci/mmol) and [3H] 3-O-methyl-D-glucose (25.5 Ci/mmol)] was obtained from PerkinElmer (Waltham, MA, USA). pBluescript ii KS(+) plasmids with open reading frames (ORFs) of human SGLT1 (OHu22190 NM_000343) and GLUT2 (OHu20407 NM_000340.2) were obtained from Genscript Biotech (Piscataway, NJ, USA). Restriction enzymes and T7 transcription materials were obtained from New England Biolabs (Ipswich, MA, USA). All other chemicals for use in glucose uptake studies were obtained from Sigma-Aldrich (St. Louis, MI, USA) unless otherwise noted. Isolated *Xenopus laevis* ovaries were purchased from Xenopus-1 (Dexter, MI, USA). The standard Modified Barth's Saline (MBS, contained (in mmol l^−1^) 88 NaCl, 1 KCl, 1 MgSO_4_, 5 HEPES, 2.5 NaHCO_3_, 0.7 CaCl_2_·2H_2_O, pH 7.4) was prepared in our lab following standard procedures. When necessary, MBS was sterilized by vacuum bottle-top filters (EMD MilliporeTM SteritopTM sterile vacuum bottle-top filters, ThermoFisher, Waltham, Massachusetts, USA) and supplemented with sodium pyruvate (1 mM), 1 mg ml^−1^ penicillin-streptomycin (Gibco, Long Island, NY, USA) per mL and 50 μg gentamicin per mL for long term storage of isolated oocytes.

### Waffle Formulation

Four waffles were formulated to provide different amounts of BG but the same amount of available carbohydrate per serving while maintaining consistency in fat, protein, and insoluble dietary fiber. Waffles containing BG were made from whole barley flour (6.1% BG, 67.8% available carbohydrate, 14.9% total dietary fiber, 14.9% protein, and 2.6% oil) or BG rich barley flour (12.2% BG, 48.8% available carbohydrate, 29.0% total dietary fiber, 16.4% protein, and 4.3% oil). Control waffles were made from all-purpose wheat flour (Robin Hood). For our clinical trial we tested 2, 4, and 6 g BG per 30 g available carbohydrate, but in the present *in vitro* study half of each treatment was used for testing in *in vitro* digestion model (1 g, 2 g, 3 g BG per 15 g available carbohydrate). Wholemeal barley was used to achieve the 1.2 g BG per 15 g available carbohydrate treatment and the 2 and 3 g BG treatments were made by substituting increasing amounts of the BG rich milling fractions. Commercial oat hull fiber (high in insoluble fiber) was supplied by Grain Millers, Inc. and added to the formulations as necessary to equalize insoluble dietary fiber as closely as possible across the treatments. Canola oil, egg white, and skim milk amounts were adjusted to equalize fat and protein and water was varied to maintain a functional batter consistency. The control made with all-purpose wheat flour was formulated to match as closely as possible the available carbohydrate, insoluble dietary fiber, protein, and oil content of the BG treatments. A second control was included to represent a typical waffle recipe that would commonly be available to consumers and therefore did not match the treatments in macronutrient composition or serving size with the exception of delivering the same amount of available carbohydrate. All waffle treatments and control formulations are shown in Table 1.

**Table 1:** Nutrient composition of waffle treatments, based on a single serving.

**Waffle type**	**Serving size (g, as is basis)**	**Available carbohydrate**	**Beta-glucan**	**Insoluble dietary fiber**	**Soluble dietary fiber**	**Total dietary fiber**	**Protein**	**Oil**
1 g BG	77	14.54	1.21	4.54	1.64	6.18	6.91	3.76
2 g BG	89	15.14	2.06	4.27	2.60	6.86	7.01	3.83
3 g BG	89	14.28	2.96	4.38	3.44	7.82	7.14	3.60
Control-matching	77	15.18	0.06	5.18	0.39	5.57	7.01	3.77
Control-typical recipe	38	14.98	0.06	0.45	0.38	0.83	3.47	3.48

### TIM-1 Gastro-Intestinal Model

The computer controlled dynamic multi-compartmental TIM-1system (TNO, Zeist, Netherlands) was used to digest waffles *in vitro* by following a fed state protocol to mimic digestion in healthy young adults. The system has been described in detail and validated by its developer (22). The system simulates conditions in the upper gastrointestinal digestion system and was operated using the “water fed state” conditions presented in Table 2. Prior to feeding the system with waffles, the gastric compartment contained 10 g of gastric start residue [pH 1.7, 5 g hydroxypropylmethylcellulose (0.4%)] and 5 g gastric enzyme solution (pepsin 4,800 U/ g; α-amylase 47 U/ g and lipase 20 U/ g), the duodenum contained 55 g duodenal start residues (15 g pancreatin solution (7%), 30 g bile solution (4%), 2 mg trypsin and 15 g small intestine electrolyte solution), and both the jejunum and ileum contained 115 g intestinal electrolyte solution (pH 7.4) each.

**Table 2:** The “water fed state” operating conditions of TIM-1 digestion system used to digest waffles.

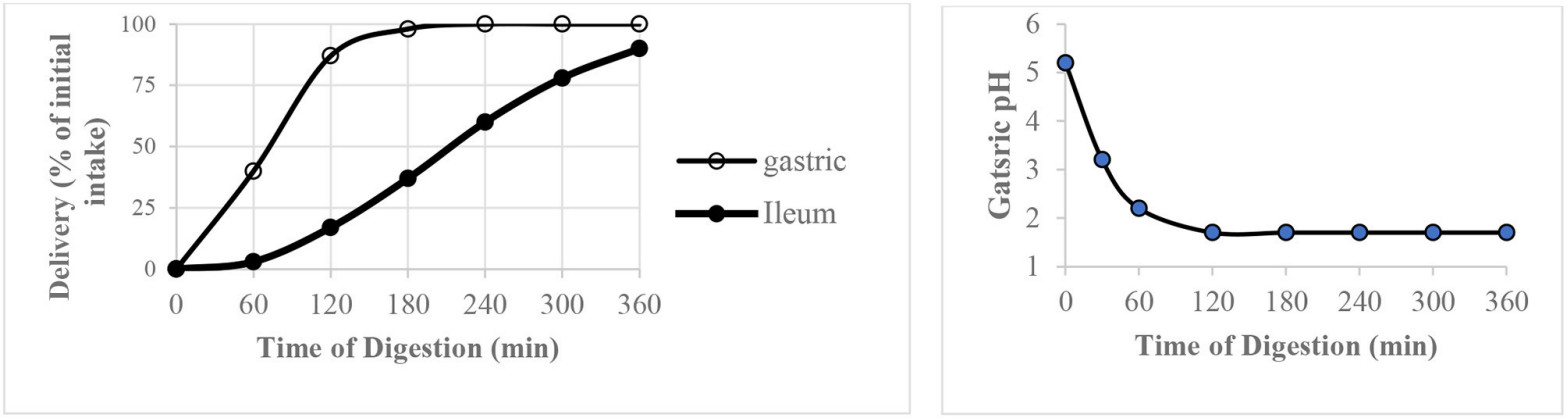	
**Parameters of *in vitro* digestion**	
**Gastric compartment**	
pH	5.7 at T_0_ to 1.7 at T_360_ see chart above
Volume	300 mL
Secretions	520 U/ min pepsin, 2 U/ min lipase, and 5 U/ min amylase
Time of half emptying	T_1/2_ = 70 min
β coefficient	2.5
**Duodenal compartment**	
pH	~ 6.2
Volume	55 mL
Secretions	Bile (20 mg/min for the first 30 min after which 10 mg/ min), pancreatic juice (80 mg/ min) and Sodium bicarbonate solution (as required)
**Jejunal compartment**	
pH	~7.4
Volume	115 mL
Secretions	Sodium bicarbonate solution (as required)
**Ileal compartment**	
pH	~7.4
Volume	115 mL
Secretions	Sodium bicarbonate solution (as required)
Time of half emptying	T_1/2_ = 220 min
β coefficient	2.5

Waffles were mixed with ~200 mL tap water (37°C) and homogenized using a kitchen hand blender. The pH of the food was adjusted to 5.5 using 1 M HCl after which 15 mg porcine pancreatic α-amylase (1,500 units/mg) was added to the food and volume adjusted to 290 mL with water. The food (37°C) was fed to TIM-1 system and digestion was initiated at ~5 min from the time α-amylase was added to the food. Waffles were digested for 360 min and jejunal and ileal dialysis fluids were changed every 60 min. The dialysis fluids collected were kept at −40°C until the day of sugar analysis. Cumulative glucose released at a time point was calculated as the sum of the sugar content in the jejunal and ileal dialysis fluids at a sampling point.

### Glucose Determination on TIM-1 Dialysates Using Acid Hydrolysis

Dialysate aliquots collected from the jejenum and ileum were frozen in 2 mL microfuge tubes until ready for analysis. Samples were defrosted and centrifuged at 9,000 × g for 2 min. 200 μL of the supernatant was pipetted to a 2 mL microfuge tube containing 200 μL 2.6 M HCl and placed in a boiling water bath for 1 h. Samples were cooled to room temperature and 50 μL was pipetted into a 10 mL glass tube containing 50 μL 1.3 M HCl. Three mL of GOPOD reagent (D-Glucose Assay kit, K-GLUC, Megazyme, Ireland) was added to the tube and incubated at 50°C for 20 min. Absorbance was read at 510 nm on a spectrophotometer (Ultrospec 3000, Pharmacia Biotech, Sweden). The amount of glucose in the aliquot was calculated against the absorbance of the external glucose standard (1 mg/ mL) which was used to calculate how much glucose was in the dialysate.

### Determination of Waffle Viscosity

BG viscosity was determined by an *in vitro* digestion method, by adding digestive enzymes to the waffle samples and incubated with stirring in a Rapid Visco Analyser (RVA) 4500. The amount of cooked waffle to weigh into the canister was based on 4.1 g dry solids, which was added to 24.487 mL of 20 mM NaH_2_PO_4_ + 10 mM NaCl buffer, pH 6.9 less the moisture amount in the cooked waffle, along with 63 μL salivary α-amylase (Sigma A1031; 220 U/mL in 2.5 mM CaCl_2_), 150 μL pepsin (Sigma P7012; 1,130 U/mL in 0.9% NaCl), and 300 μL pancreatin (Sigma P7545; 0.5 mg/mL in 20 mM NaH_2_PO_4_ + 10 mM NaCl buffer, pH 6.9). The RVA paddle speed was set to 480 rpm for 10 s, followed by 160 rpm for a total of 2 h, with the temperature maintained at 37°C. The final slurry viscosity was recorded at 2 h. A subsample of the slurry was transferred to microfuge tubes and centrifuged at 9,000 × g. The supernatant viscosity was measured on a DHR-2 rheometer (TA Instruments, New Castle, DE, USA) fitted with a 4°, 40 mm geometry. The Pelletier plate was set to 37°C, and a flow ramp method with an initial shear rate of 0.1/s, increasing to 100/s logarithmically over 2 min was used. The viscosity at 30/s shear rate was determined.

### Effect of BG on Intestinal Brush Border α-glucosidase Activity

The effect of BG on intestinal brush border α-glucosidase activity was determined according to Malunga et al. (23). Briefly, intestinal α-glucosidase was extracted from rat intestinal acetone powder with sodium phosphate buffer (pH 6.9, 0.1 M). Two barley BG (Megazyme) with average molecular weight of 650,000 (BG-HMW) and 229,000 (BG-LMW) were used to create solutions of different viscosity. BG was added to water and brought to 90°C whilst stirring continuously on a magnetic stirrer until the mixture was clear. The mixture was diluted to 2, 4, and 6 mg / mL with sodium phosphate buffer (pH 6.9, 0.1 M) after cooled to room temperature. Later, 100 μL BG solution or buffer (control) was mixed with 50 μL rat intestinal α-glucosidase in a screw cap test-tube. The mixture was incubated at 37°C for 5 min and digestion was initiated by adding 50 μL of maltose (60 mg/ mL). Enzyme activity was stopped after 30 min by heating at 95°C for 10 min. The mixture was centrifuged (10,000 × g, 4°C, 10 min). The glucose released was analyzed using the Megazyme GOPOD glucose test kit.

### Preparation of cRNA for Oocyte Expression

DH5α cells containing SGLT1 and GLUT2 ORFs were propagated and the plasmid extracted using the plasmid miniprep kit (Qiagen, Hilden, Germany) and linearized with XbaI (New England Biolabs, Canada (NEB) following the manufacturer's guidance. The *in vitro* transcription of the capped mRNA (cRNA) was done using the HiScribe™ T7 ARCA mRNA Kit (with tailing) (NEB) and was later purified using E.Z.N.A.® MicroElute RNA Clean Up Kit (Omega Bio-Tek, Norcross, GA, USA) and eluted in DEPC treated water.

### Preparation of Oocytes

For the oocyte experiments, oocytes were extracted from isolated ovaries. The removal of the ovaries were performed by the supplier (Xenopus 1) and the isolated ovaries were shipped to us later. Isolated *Xenopus laevis* ovaries received from the supplier were incubated in Ca^2+^-free MBS buffer containing 55 mg/mL collagenase type IV (Gibco) and gently agitated at room temperature for defolliculation. After 30 min, the oocytes were washed three times with standard MBS and allowed to recover overnight at 18°C in sterile MBS containing gentamycin (50 μg/ml) and sodium pyruvate (1 mM) with daily medium change until experiments were performed.

### Injection of Oocytes

The oocytes were then injected with 36.8 nl of SGLT1 or GLUT2 cRNA (500 ng/μl) after being rested for 24 h using the Nanoject III Programmable Nanoliter Injector (Drummond Scientific, Broomall, PA, USA). Some oocytes (labeled SHAM) were injected with DEPC treated water (36.8 nl) instead of GLUT2 or SGLT1 cRNA as a negative control. The injected oocytes were incubated in MBS at room temperature for 48 h before conducting glucose uptake studies.

### Glucose Uptake Studies in GLUT2 or SGLT1 Injected Oocytes

Barley BG (Megazyme) was added to water and brought to 90°C whilst stirring continuously on a magnetic stirrer until the mixture was clear. The mixture was cooled to room temperature and diluted to 2, 4, and 6 mg / mL with standard MBS buffer. Two types of barley BG were used namely a) BG with an average molecular weight of 650,000 (BG-HMW) and b) BG with an average molecular weight of 229,000 (BG-LMW). Glucose uptake studies were conducted in MBS transport buffer containing 125 pmol radiolabeled glucose and BG (0, 1, 2, and 3 mg / ml). Oocytes (15) were transferred to 200 μl transport buffer and incubated for 30 min at room temperature. Transport was terminated by adding ice cold MBS in excess. The oocytes were washed four times with ice cold MBS and lysed individually in vials containing 200 μl sodium dodecyl sulfate (10%). Ultima Gold scintillation cocktail (5 ml) (PerkinElmer) was added to each vial and internal radioactivity was quantified by liquid scintillation spectrometry as counts per minute (CPM). [^3^H] 2-deoxyglucose and [^3^H] 3-O-methyl-D-glucose were used as substrate in glucose transport experiments involving oocytes expressing GLUT2 and SGLT1, respectively.

### Statistics

TIM-1 experiments were run in duplicate, α-glucosidase experiments were done in sextuplicate, and glucose uptake experiments were done using 15–20 oocytes per treatment. The effect of BG on the outcome variables was tested using Analysis of Variance with *p* < 0.05 as the cut off for significance. Differences among means were determined using Tukey honest significant difference method. Statistical analysis was performed using SAS software (version 9.4, SAS Institute, Inc, Cary, NC).

## Results

### Waffle Viscosity

Table 3 shows the *in vitro* viscosity of the formulated waffles. The viscosity of formulated waffles ranged from 0.008 to 2.6 Pa.s. The waffle with the highest concentration of BG (3.0 g) had the highest viscosity among all the waffles. The viscosity of the waffles increased with higher BG content in the waffles. Addition of oat hull or insoluble fiber did not affect the viscosity of the wheat-based waffles.

**Table 3:** *In vitro* viscosity of waffles containing different BG concentrations.

**Waffle type**	**RVA**	**Rheometer**
	**final viscosity, cP**	**viscosity @ 30/s, Pa. s**
1 g BG	796	0.223
2 g BG	2,055	1.203
3 g BG	2,663	2.556
Control – matching	276	0.008
Control – typical recipe	92	0.008

*The data represent the means of n = 2 waffles*.

### The *in vitro* Carbohydrate Digestibility of Waffles Using TIM-1 Dynamic Stomach Model

**Figure 1:**
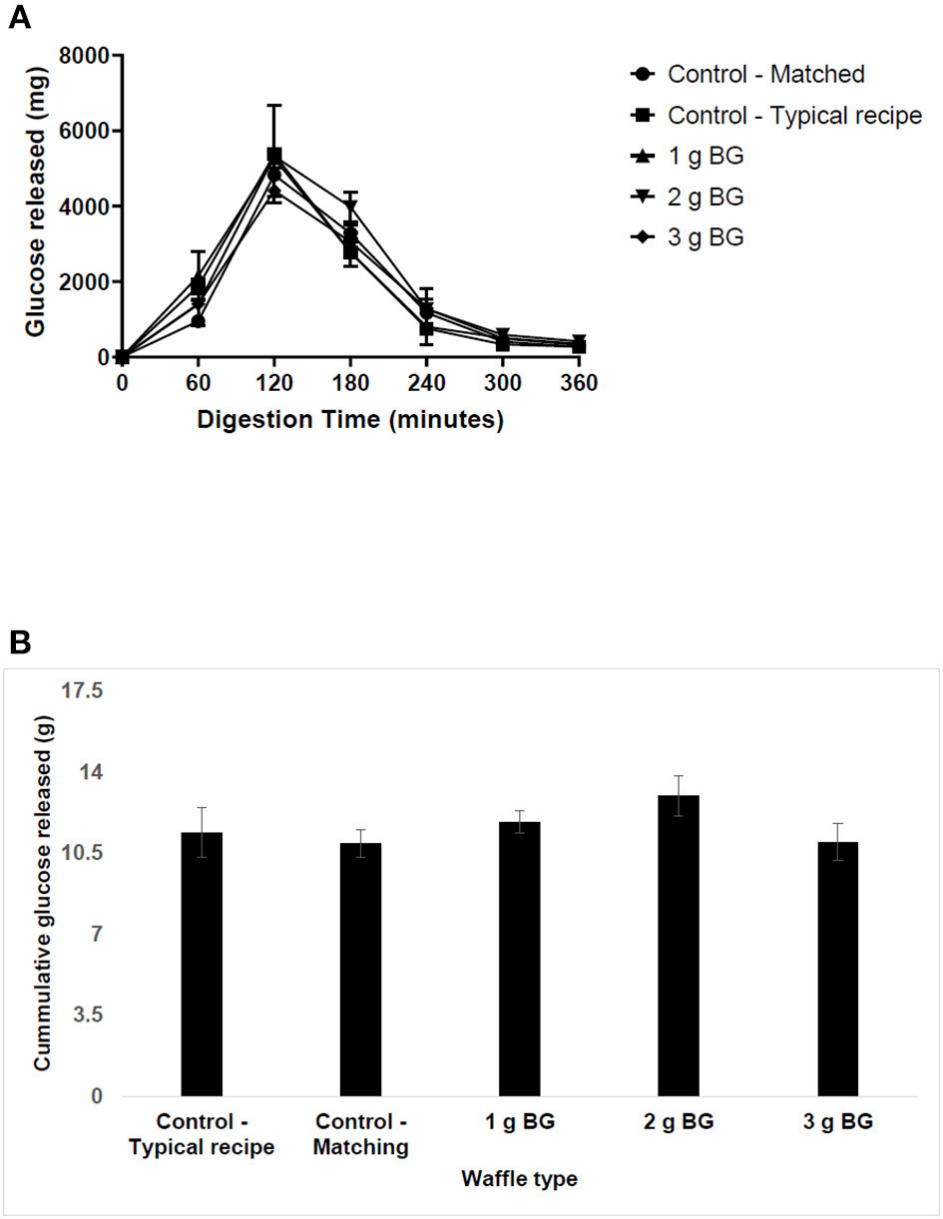
The *in vitro* digestibility of waffle carbohydrates using TIM-1 digestion model. Waffles containing ~15 g of available carbohydrate were fed to the TIM-1 system and the dialysates were collected from jejunum and ileum compartments every 60 min for glucose analysis after acid hydrolysis. The data represent mean ± SD (*n* = 2). **(A)** The release of glucose in hydrolysates over time. **(B)** Total carbohydrate released over 360 min of digestion.

The first set of experiments examined the effect of BG concentration on saliva and pancreatic α-amylase activity. The results of carbohydrate digestibility in the TIM-1 digestion system are presented in Figure 1. The peak of carbohydrate digestibility was reached at 120 min for the typical wheat-based waffles and the digestion was almost complete by 300 min. Addition of more protein or insoluble fiber or oil to the waffles, for example in the second control (matched) waffle, slowed the release of hydrolysed carbohydrates compared to the typical waffle (Figure 1A) i.e., the peak glucose released was reduced from ~5,371 to ~4,829 mg. The second control waffle (matched) with no soluble dietary fiber but set a benchmark for insoluble dietary fiber, protein, oil, and available carbohydrate for the barley waffles.

Comparing the digestion profiles of the matched wheat-based waffle and a barley waffle delivering 1.2 g BG, the results indicate that addition of BG did not affect the digestion profile except at 60 min. For example, the amount of glucose released at 60 min increased from ~950 to ~1,980 mg for waffle containing 1 g BG. This suggests that barley flour may have a higher amount of rapidly available carbohydrate compared to wheat. Increasing BG concentration in waffles to 2 or 3 g did not change their digestion profile compared to the matched wheat-based waffles. Figure 1B shows the cumulative amount of hydrolysed sugars over 360 min digestion time. The total amount of hydrolysed sugars was highest for the 2 g BG waffle and lowest for the 3 g BG waffles but not significantly different compared to all the other waffle types tested in this study.

### Effect of Barley BG on the α-Glucosidase Activity

**Figure 2:**
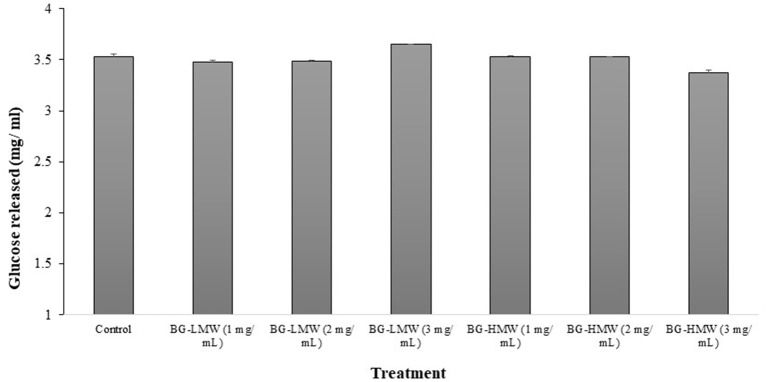
Effect of BG on mammalian intestinal alpha-glucosidase activity. Maltose (30 mg/mL) was mixed buffer (control) or buffers containing different amounts barley BG (BG-HMW: 650,000 average molecular weight; BG-LMW: 229,000 average molecular weight). Data are mean + standard deviation (*n* = 6). No statistical difference (*p* < 0.0001) was observed between means of treatment groups.

The effect of barley BG on the activity of mammalian intestinal α-glucosidase is presented in Figure 2. The concentration of glucose released after 30 min of incubation ranged from 3.35 to 3.65 mg/ mL. Figure 2 shows that the mean concentration of glucose released in the control sample was 3.53 mg/ mL. Addition of BG did not significantly affect the concentration of glucose released regardless of BG molecular weight nor concentration. These results suggest that barley BG does not affect the activity of α-glucosidase at the tested concentration.

### Effect of Barley BG on the Glucose Uptake in Oocytes Expressing Human GLUT2

**Figure 3:**
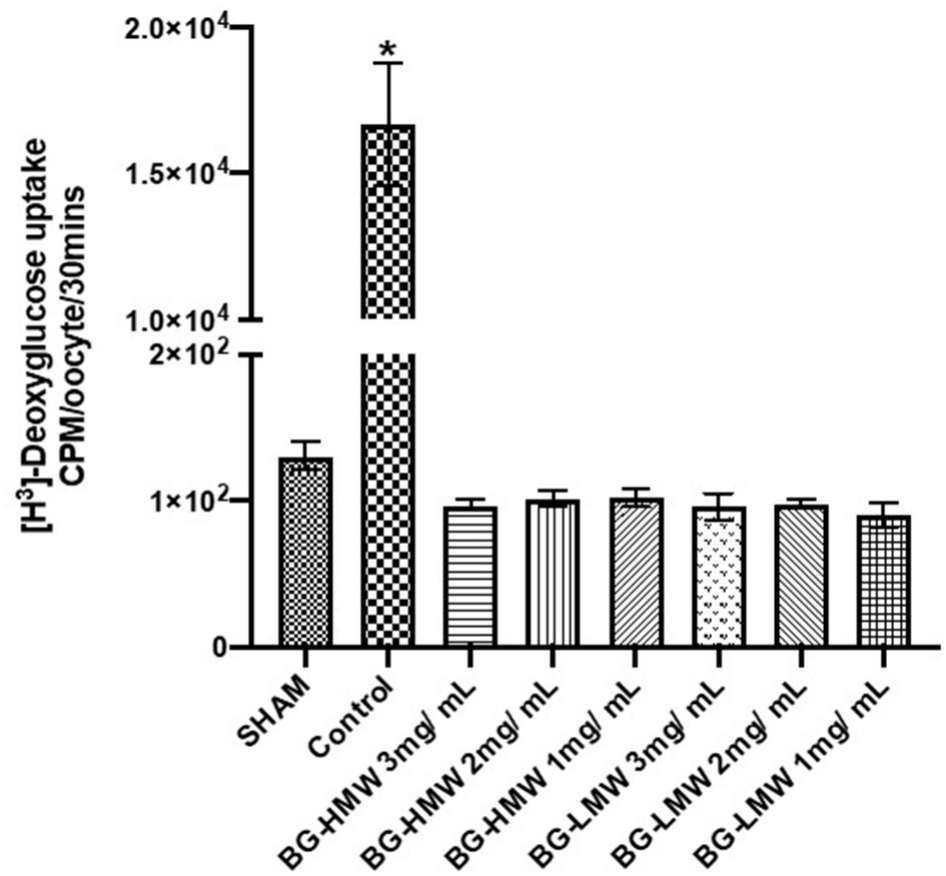
Effect of BG on glucose uptake in oocytes expressing human GLUT2. Oocytes expressing human GLUT2 and oocytes injected with water (SHAM, negative control) incubated in buffer containing radiolabeled glucose only (control) or buffers containing different amounts barley BG (BG-HMW: 650,000 average molecular weight; BG-LMW: 229,000 average molecular weight). Data are mean + SEM (*n* = 12–15 oocytes per treatment). *Significantly different from all other treatment groups *p* < 0.0001.

Oocytes expressing human GLUT2 were incubated in buffer containing radiolabeled glucose only (control) or buffers containing different amounts of barley BG (Figure 3). In the absence of GLUT2 (Sham), oocytes did not transport glucose as expected (Figure 3). The glucose uptake in GLUT2 expressing oocytes was ~16,000 CPM per 30 min. Adding barley BG to the transport medium at concentrations equivalent to the waffles (1, 2, and 3 g per L gastric volume) completely inhibited glucose uptake. Figure 3 shows glucose uptake by GLUT2 in the presence of barley BG with an average molecular weight of 650,000 and that for barley BG with an average molecular weight of 229,000. Barley BG at all the tested concentrations (1, 2, and 3 mg / mL) completely inhibited the uptake of glucose mediated by GLUT2. Similarly, the inhibition potency of BG with an average molecular weight of 650,000 was not significantly different from that of BG with an average molecular weight of 229,000. Specifically, in the presence of BG (regardless of BG concentration or its molecular weight), the amount of glucose absorbed was like that of oocytes not expressing GLUT2 suggesting complete nullification of GLUT2 activity.

### Effect of Barley BG on the Glucose Uptake in Oocytes Expressing Human SGLT1

**Figure 4:**
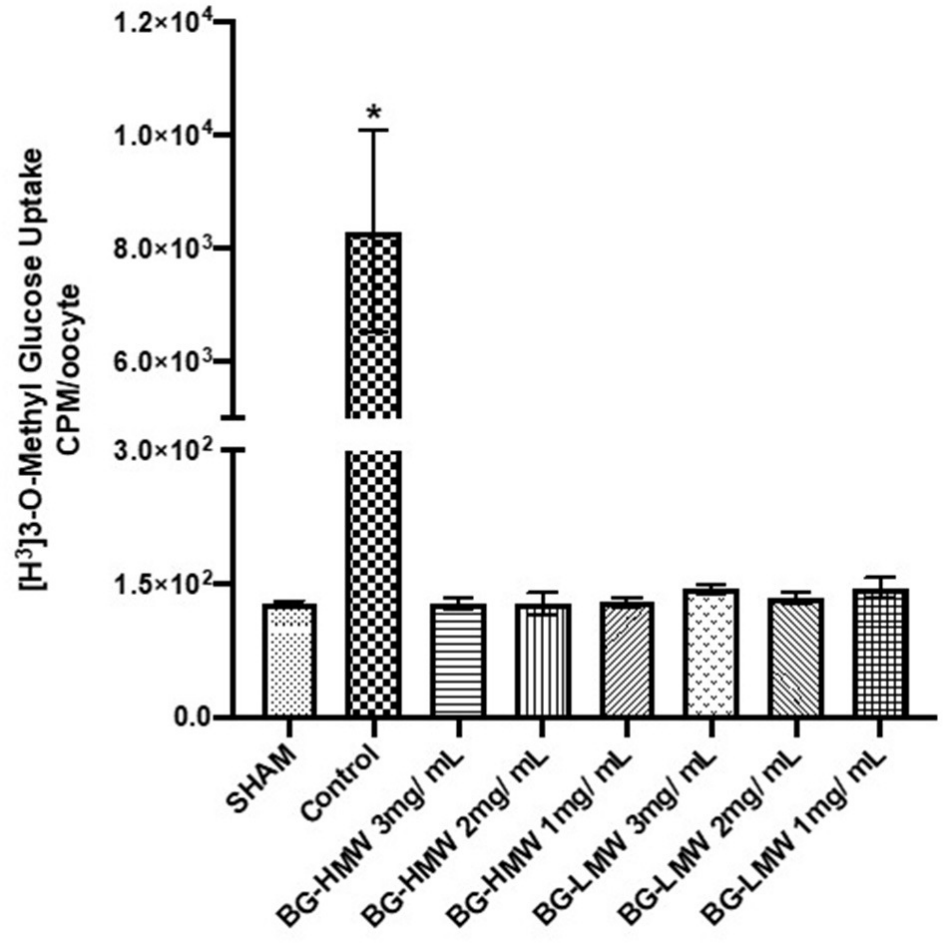
Effect of BG on glucose uptake in oocytes expressing human SGLT1. Oocytes expressing human SGLT1 and oocytes injected with water (SHAM, negative control) were incubated in buffer containing radiolabeled glucose only (control) or buffers containing different amounts barley BG (BG-HMW: 650,000 average molecular weight; BG-LMW: 229,000 average molecular weight). Data are means ± SEM (*n* = 10–12 oocytes per treatment). *Significantly different from all other treatment groups *p* < 0.0001.

The effect of barley BG on the glucose uptake in oocytes expressing human SGLT1 is presented in Figure 4. Glucose uptake in SGLT1 expressing oocytes ranged from ~120 to ~10,000 CPM per 30 min. The results show that BG at all tested concentrations (1, 2, and 3 mg/mL) significantly reduced the uptake of glucose mediated by SGLT1. Pairwise comparison of the mean glucose uptake between the 1, 2, and 3 mg BG per mL showed no significant difference within BG molecular weight types.

## Discussion

It is generally thought that the mechanism through which BG delays carbohydrate digestibility and glucose uptake is related to its ability to form viscous solutions (24). It has been hypothesized that the resultant high viscosity limits the interaction between digestive enzymes and their substrate as well as that of nutrients and nutrient transporters leading to a reduction in post-prandial glucose concentration (5). However, our results suggest for the first time that BG does not lower blood glucose because of its high viscosity but rather through direct interaction with intestinal brush border enzymes and nutrient transporters. In our study, the developed waffles had substantially different viscosities (0.008, 0.223, 1.203, and 2.556 Pa. s) and yet their starch digestibility through the TIM-1 system did not vary significantly. Viscosity of BG is dependent on its concentration and molecular weight average (10). Therefore, BG of varying molecular weights and concentrations were used for α-glucosidase and glucose uptake studies to generate experimental mediums of different viscosities. Higher concentrations and molecular weights generate more viscous BG solutions (10). We found that the effect of the BG on the activities of α-glucosidase, GLUT2, and SGLT1 was not concentration or molecular weight dependent. These findings do suggest that BG's effect on the activities of α-glucosidase, GLUT2, and SGLT1 are not explained by viscosity. BG at dietary concentrations exhibit non-Newtonian fluid behavior where the polysaccharides may thin out under high shear stress (13). Thus, it is plausible that the BG apparent viscosity is counteracted by intestinal peristalsis.

The first step in carbohydrate assimilation involves the hydrolysis of starch by α-amylase present in the saliva and pancreatic juice. Barley waffles containing 1.2, 2.0, and 3.0 g BG per 15 g available carbohydrate were fed to the TIM-1 system to study the effect of BG on starch digestibility. The EFSA panel on Dietetic Products and Allergies recommends taking 4 g BG per 30 g available carbohydrate to lower post-prandial blood glucose concentration (15). The amount fed to the TIM-1 system in this study was equivalent to 2.4, 4, and 6 g BG per 30 g available carbohydrate. Thus, we anticipated that at least waffles containing 4 and 6 g BG should significantly reduce the amount of hydrolysed sugars in the dialysate. In fact, our group used these same waffle formulations in a clinical trial (Clinicaltrial.gov Identifier: NCT02367989) and found that all three BG treatments significantly lowered post-prandial glucose concentrations (16). The lack of significant difference in the *in vitro* digestibility profiles of waffles suggest that BG does not affect amylase activity in the gastrointestinal tract as hypothesized previously. This finding is consistent with a previous study with a different soluble fiber, arabinoxylan, where amylolysis of starch was not affected by addition of arabinoxylan *in vitro* (23).

It is possible that the lack of difference in starch hydrolysis could be a consequence of the difference in BG and/or starch source used in this study. Waffles were made from flour constituting different grain fractions to achieve a desired nutrient composition. The solubility of BG may vary depending on its source and botanical fraction (25). However, the waffle viscosity measurements corresponded with the increase in BG content demonstrating that BG solubility was not affected based on the source and botanical fraction. Additionally, starches obtained from different botanical sources may have different digestibility potential as they may vary in their amylose to amylopectin ratio (26). If this were true, the numerical difference in the amount of hydrolysed sugars released between barley waffles made from whole barley flour (BG 1 g), an equal blend of whole barley flour with the BG rich fraction (2 g) and that made from the BG rich fraction alone (BG 3 g) would be linear. Our results did not show a linear response in the amount of hydrolysed sugars released between barley waffles further strengthening the case that source and botanical fraction did not affect the observed results. Starch digestibility is also affected by the level of gelatinisation attained during the thermal processing (27). The gelatinization potential of starch can be affected by BG which competes with starch for the available water (28). However, our results suggest that the carbohydrate digestibility of waffles was neither affected by the difference in the source of starch nor by the dose of BG as evidenced by lack of clear observable pattern in the amount of glucose released numerically. Thus, we concluded that the lowered post-prandial blood glucose observed *in vivo* after taking a BG rich meal is not a consequence of BG influencing the amylolysis of starch.

The products of starch hydrolysis by α-amylase together with the inherent disaccharides or oligosaccharides present in foods are hydrolysed by the intestinal brush border maltase-glucoamylase and sucrase-isomaltase to monosaccharides prior to their absorption. Most of the available pharmaceutical molecules for management of diabetes target the intestinal brush border α-glucosidase activity. Therefore, we examined the effect of BG on intestinal brush border α-glucosidase activity. The results indicated that brush border α-glucosidase activity was not affected by BG regardless of BG's molecular weight or concentration. Similarly, arabinoxylan obtained from wheat could only inhibit α-glucosidase activity when feruloylated (23). This further suggests that viscosity of digesta generated by soluble fiber may not influence the digestibility of carbohydrates.

The last step in the intestinal carbohydrate digestion involves transportation of monosaccharides across the intestinal epithelia. Transport of monosaccharides in the intestine is mediated by passive transport through GLUT2 and GLUT5 and by active transport through SGLT1 (19, 20). Both SGLT1 and GLUT2 activities were significantly inhibited by BG at all concentrations tested in this study. Our results suggest that the lowered post-prandial blood glucose concentration in the presence of BG maybe a consequence of BG's ability to inhibit intestinal glucose uptake *via* SGLT1 and GLUT2 transporters. SGLT1 is expressed on the apical side of the intestine and transports most of the glucose when the glucose concentration of the intestinal digesta is low (29). On the other hand, GLUT2 is mostly found on the basolateral side and is responsible for exporting glucose to blood. As the concentration of glucose increases, usually after consumption of available carbohydrate rich meals, GLUT2 is also expressed on the apical side and works together with SGLT1 to mediate glucose uptake (29, 30). In a previous study, Abbasi et al. reported that BG reduced the glucose uptake in IEC-6 cells by suppressing the expression of GLUT2 and SGLT1. Our results however suggest that glucose uptake inhibition by BG is achieved through substrate-inhibitor-transporter interaction. Both SGLT1 and GLUT2 expression on the intestinal brush border layer is dependent on the luminal glucose concentration. It is possible that the reduced mRNA expression observed in the previous study was a consequence of reduced glucose uptake due to BG interaction with the pre-expressed GLUT2 and SGLT1. Glucose uptake in our study used pre-expressed SGLT1 or GLUT2. Therefore, it is reasonable to propose that BG affects glucose uptake by inhibiting the activities of SGLT1 and GLUT2, and consequently SGLT1 and GLUT2 mRNA expression is suppressed.

## Conclusion

This study has indicated that barley BG attenuates post-prandial glycemic response by influencing the activities of GLUT2 and SGLT1 but not amylolysis of starch and α-glucosidase activity. The glucose transporter activity was inhibited in the presence of barley BG. The effect of BG on GLUT2 and SGLT1 activities was not a consequence of BG's viscosity but rather a direct interaction between BG and membrane active proteins. We have also demonstrated that differences in BG viscosity may not affect α-amylase, α-glucosidase, GLUT2, and SGLT1 activities. Thus, further molecular studies are required to understand the interaction between BG and membrane active proteins.

## Data Availability Statement

The original contributions presented in the study are included in the article/supplementary material, further inquiries can be directed to the corresponding author/s.

## Author Contributions

LM, NA, and ST contributed to conception and design of the study. LM and HZ performed experiments and statistical analysis. LM wrote the first draft of the manuscript. LM and ST wrote sections of the manuscript. LM, NA, HZ, HB, and ST contributed to manuscript revision, read, and approved the submitted version. All authors contributed to the article and approved the submitted version.

## Conflict of Interest

The authors declare that the research was conducted in the absence of any commercial or financial relationships that could be construed as a potential conflict of interest.
